# Identifying vulnerable mother-infant dyads: a psychometric evaluation of two observational coding systems using varying interaction periods

**DOI:** 10.3389/fpsyg.2024.1399841

**Published:** 2024-06-24

**Authors:** Helen Sharp, Silia Vitoratou, Heather O’Mahen, Laura Bozicevic, Miriam Refberg, Chloe Hayes, Jessica Gay, Andrew Pickles

**Affiliations:** ^1^Department of Primary Care and Mental Health, Institute of Population Health, University of Liverpool, Liverpool, United Kingdom; ^2^Department of Biostatistics and Health Informatics, Institute of Psychiatry, Psychology and Neurosciences, King’s College London, London, United Kingdom; ^3^Washington Singer Laboratories, University of Exeter, Exeter, United Kingdom

**Keywords:** mother-infant interaction, reliability, predictive validity, infant mental health, attachment

## Abstract

**Introduction:**

Clinical services require feasible assessments of parent-infant interaction in order to identify dyads requiring parenting intervention. We assessed the reliability and predictive validity of two observational tools and tested whether briefer forms could be identified which retain acceptable psychometric properties over short observation periods.

**Methods:**

A stratified high-risk community sample of 250 mother-infant dyads from The Wirral Child Health and Development Study completed 7-min play-based interaction at 6–8 months. Film-footage was independently coded by two trained raters using PIIOS and NICHD-SECCYD systems. Incremental predictive validity was assessed from 3, 5 and 7 min observation to attachment outcomes (Strange Situation; 14 months) and infant mental health (BITSEA; 14 and 30 months).

**Results:**

Excellent inter-rater reliability was evident at code and subscale level for each tool and observation period. Stability of within-rater agreement was optimal after 5 min observation. ROC analysis confirmed predictive (discriminant) validity (AUCs >0.70) to top decile age 2 mental health outcomes for PIIOS total score and a brief 3-item composite from NICHD-SECCYD (sensitivity, intrusiveness, positive regard; NICHD-3), but not to attachment outcomes. Logistic regression showed dyads rated at-risk for externalizing problems using NICHD-3 were also at significantly higher risk for insecurity at 14 months (OR = 2.7, *p* = 0.004).

**Conclusion:**

PIIOS total and NICHD-3 ratings from 5 min observation are both reliable and valid tools for use in clinical practice. Findings suggest NICHD-3 may have greater utility due to its comparative brevity to train and code, with suitability for use over a broader developmental time frame (3–24 months).

## 1 Introduction

Worldwide, numerous community and clinical studies have shown that the quality of early parent–child interaction is a significant predictor of later child development, including attachment security (Bakermans-Kranenburg et al., 2004; McElwain and Booth-LaForce, 2006; Mills-Koonce et al., 2007; Wright et al., 2018), socio-emotional development (Murray et al., 2007; Wright et al., 2018; Cooke et al., 2022) and cognitive development (NICHD Early Child Care Research Network and Duncan, 2003; Valcan et al., 2018). This body of research underpins government initiatives such as the UK Best Start for Life Programme: A Vision for the first 1,001 Critical Days (Department of Health and Social Care, 2021) and the US Early Head Start Programme (2013) which emphasize the importance of supporting early infant-caregiver relationships at the earliest opportunity, so as to minimize adverse developmental outcomes for children. A major challenge then, for clinicians and other perinatal and early years professionals, is how best to reliably identify caregiver-infant dyads that most require intervention. Observational tools to assess parenting are widely agreed to be a gold standard approach providing an objective assessment and generating different information to self-reports from parents which are subject to informant biases (Corcoran and Fischer, 2013). However, in research contexts observation periods are typically longer than is feasible in clinical practice, and coding requirements are often more intensive than practicable. We report findings from a study designed to test the reliability and predictive validity of two observational parent-infant interaction assessment tools, over incremental brief observational periods, to establish their potential for use routinely by clinicians in the first year of life.

Compromised parenting at this early stage in development is seen particularly, though not exclusively, in the context of parental mental health problems, and is often indexed by lower maternal sensitivity to infant signals, higher withdrawn or intrusive behaviors, higher negative regard and/or lower levels of positive regard (warmth) expressed towards the infant (e.g., Nicol-Harper et al., 2007; Feldman et al., 2009; Murray et al., 2010; Azak and Raeder, 2013). For instance, studies of postnatal depression have shown lowered behavioral responsivity and sensitivity to infant signals (Stanley et al., 2004) and fewer signs of overt affection (Herrera et al., 2004). These disturbances are most evident when depression is chronic and/or severe (Campbell et al., 1995; Netsi et al., 2018) and are strongest in the context of socio-economic deprivation (Taraban and Shaw, 2018). Importantly, ameliorating depression alone has not been shown to sufficiently improve mother-infant interactions (Poobalan et al., 2007). Longitudinal prospective studies provide evidence that early parenting is an important mediator or moderator of adverse effects of perinatal mental health problems on child cognitive and social–emotional development (e.g., NICHD Early Child Care Research Network, 1999; Campbell et al., 2004; Milgrom et al., 2004; Tomlinson et al., 2005; Murray et al., 2010; Goodman et al., 2020), hence there is an additional focus for clinicians working with parents in the perinatal period on assessment of early parenting and use of evidence based interventions to ameliorate difficulties.

The economic argument for identifying troubled dyads and intervening to improve early parent-infant relationships is strong. For instance, in the context of perinatal mental health problems perinatal depression, anxiety and psychosis carry a total long-term cost to society of about £8.1 billion for each one-year cohort of births in the UK (Bauer et al., 2014) but importantly nearly three-quarters (72%) of these costs are related to adverse lifetime impacts on the child rather than the mother. Similarly, in the US a recent public health report estimated the costs of not treating perinatal mental health conditions was $14.2 billion in 2017, with one third of the costs being attributed to adverse child outcomes in the first five years of life (Luca et al., 2020).

Internationally, clinicians require assessment tools to identify those in need of intervention with proven predictive validity, a robust evidence-base and that can feasibly be implemented in busy clinical practice. However, many observational tools either have unknown predictive validity, have been evaluated in research studies using lengthy observation periods (typically 10–30 min; Mesman and Emmen, 2013), or are deemed too time-intensive to code in routine clinical practice (Royal College of Psychiatry, 2018). There is also a wide range of observational scales to choose from. For instance, Mesman and Emmen’s (2013) systematic review identified 50 observational measures designed to assess parental sensitivity. They highlighted eight measures with the most robust research pedigrees. These measures included a coding scheme developed for the National Institute of Child Health and Human Development study of Early Child Care and Youth Development (NICHD-SECCYD) (Owen, 1992) and Crittenden’s CARE Index (Crittenden, 2001). In this study we focused on evaluating the psychometric properties of two candidate scales, selected for different reasons. The Parent Infant Interaction Observation Scale (PIIOS) (Svanberg et al., 2013) was developed in the UK and cross-sectionally validated against the Maternal Sensitivity subscale of the Crittenden Care Index as a screening tool for universal use. Training on PIIOS had already been rolled out in UK specialist perinatal mental health teams on the basis of this. For this scale, three to 4 min of observation are recommended. However, its predictive validity to later child outcomes was unknown and required testing. By contrast the NICHD-SECCYD system, developed in the US, has repeatedly been shown to have predictive validity to later socio-emotional outcomes including attachment security (e.g., McElwain and Booth-LaForce, 2006; Mills-Koonce et al., 2007; Leerkes et al., 2009; Birmingham et al., 2017), but in research contexts observation periods are typically longer (>10 min) which may limit its routine use in clinical practice.

The full NICHD-SECCYD system (NICHD) and PIIOS systems are similar in that they each involve coding multiple dimensions of parent-infant interaction and both aim to capture parental sensitive-responsiveness. The NICHD system includes ratings of 14 dimensions of parent–child interaction, though research studies have typically used the scales in a hypothesis driven manner to examine specificity of prediction from specific parenting dimensions to later outcomes of interest. Some have used a single coding dimension (e.g., global sensitivity or sensitivity to distress) and others have used subsets of codes. Commonly a 3-item composite has been used to yield a broader ‘sensitivity’ composite, using sensitivity to non-distress or global sensitivity (which includes non-distress and distress episodes during play), positive regard (warmth) and intrusiveness (reverse coded) examining prediction to later child mental health or attachment related outcomes (NICHD Early Child Care Research Network, 1997; Bakermans-Kranenburg et al., 2004; Campbell et al., 2004; Mills-Koonce et al., 2007; Birmingham et al., 2017). In contrast, for PIIOS there is a limited research literature with only one study describing the development of the measure (Svanberg et al., 2013). The manual (Svanberg, 2009) recommends calculation of a total score across 13 parent-infant interaction dimensions to index overall sensitive-responsiveness and then conversion of this score into three categorical domains (no concern, some concern, significant concern), designed to drive clinical decision making and intervention level [e.g., Universal, Universal Plus and Universal Partnership as part of the Healthy Visitor Implementation Plan within the Healthy Child Program (Department of Health, 2009, 2011)]. To our knowledge no research has yet been conducted to examine whether or not the whole scale or a subset of codes within PIIOS has utility in the prediction of later child health outcomes. Finally, since the full NICHD and the full PIIOS system each require coding 13 or 14 dimensions of interaction we were keen to test whether brief forms of each tool (formed using only a subset of codes) could be derived with sufficient reliability and predictive validity, in view of the obvious time and cost-saving implications for implementation in clinical practice.

In summary, we aimed to assess the inter-rater reliability and predictive validity of these two widely used parent-infant interaction observational assessments; The NICHD system and the PIIOS. We rated parent-infant interactions filmed when infants were between 6 and 8 months old as part of an ongoing longitudinal study of child health and development. We felt this film archive was a suitable resource since it represents a common time for assessment in clinical services, once mental health or parent-infant difficulties have come to the attention of professionals. Child outcomes were attachment security and disorganization assessed at mean age 14 months and child externalizing and internalizing problems reported by parents at mean age 14 months and 30 months. In order to determine which measure or combination of codes within each scale had optimum predictive validity and brevity, and thus clinical utility, we examined whole scale performance and item-subset performance, determined *a priori* from the research literature or from factor analytic or machine learning analyses conducted within this study. Since parents with moderate to severe mental health problems may not tolerate lengthy observation, we further tested whether lengthier observation periods incrementally improved inter-rater reliability and predictive validity to child outcomes or not, examining ratings from 3 min, 5 min and 7 min of observation.

## 2 Materials and methods

### 2.1 Sample

Participants were members of the Wirral Child Health and Development Study in which a larger (‘extensive’) general population sample (*n* = 1,233) of primiparous women was consecutively recruited at 20 weeks of pregnancy between February 2007 and October 2008 and then used to provide a stratified simple random subsample for more detailed study, the ‘intensive’ sample (*n* = 316), and both were then followed longitudinally in tandem. The focus in this report is data gathered from the intensive sample. The stratification variable, inter-partner psychological abuse reported by the women (Moffitt et al., 1997), was chosen for its known association with a variety of risk factors for early child development. All participants in the extensive sample scoring above the threshold for psychological abuse from partner-to-self or self-to-partner at 20 weeks gestation were eligible for inclusion in the intensive sample plus a random selection from those below the threshold. The intensive sample therefore comprised a higher risk community sample of first-time mothers in which 51 and 49% represented high and low risk strata, respectively. The stratification variable was effective in generating a higher risk perinatal sample, as evidenced by the fact that the mean prenatal EPDS scores (Cox et al., 1987) in the low vs. high risk strata were 6.67 (SD 3.97) vs. 9.86 (SD 4.80), Cohen’s d = 0.68, *p* < 0.001.

The intensive sample of 316 women with live singleton babies were assessed first at 20 weeks of pregnancy. Of these, 272 mother-infant dyads were later filmed during play when the infants were mean 29.1 (SD 3.1) weeks of age. Film footage from 7 dyads with two periods of 7- and 8-min play observation (14 periods) were used for training and footage from 15 dyads (30 periods of observation) were used as an initial reliability set. A total of 250 dyads were therefore available for the current study proper. Of these, 231 (92%) completed the attachment assessment at mean age 14 months, whereas 219 (88%) parents completed reports of child mental health at age 14 months and 30 months, respectively. Socioeconomic conditions on the Wirral range between the deprived inner city and more affluent suburbs, but with low numbers from ethnic minorities. The demographic characteristics of the sample are given in Table 1. Just under 40% of the sample were living in socio-economic conditions equivalent to the bottom quintile of the UK population as a whole and just 3% were from non-white British ethnicities which is representative of the local area from which participants were drawn.

**Table 1:** Sample demographics characteristics.

Demographic characteristic	*n*	*M*	SD	Range
Maternal age (years)	250	27.69	6.14	11–51
Age of child (months)				
Age 1 follow-up	231	14.12	1.68	11–20
Age 2 follow-up	219	30.86	2.32	27–42
		*%*		
Child Sex at birth	250			
Male	127	50.8		
Female	123	49.2		
Marital status	249			
Married/partnered	190	76.4		
Single/divorced/separated	59	23.6		
Ethnicity	250			
White British	241	96.4		
Other	9	3.6		
Maternal education	250			
<18 years	86	34.4		
> = 18 years	164	65.6		
SES	250			
IMD 1 most deprived	94	37.6		
IMD 2	51	20.4		
IMD 3	66	26.4		
IMD 4	16	6.4		
IMD 5 least deprived	23	9.2		
Sample stratifier	250			
Low risk	118	47.2		
High risk	132	52.8		

IMD, Socioeconomic status derived from post code data using the English Index of Multiple Deprivation [IMD (Noble et al., 2004)] and converted to quintile categories with a binary variable (1 = most deprived, 0 = all 4 other quintiles) used for analysis. The sample stratifier was inter-partner psychological abuse (see measures for details).

### 2.2 Ethical approvals

All women gave written informed consent into the Wirral Child Health and Development Study and at multiple subsequent follow-up time points. Ethical approval for the study was granted by the Cheshire North and West Research Ethics Committee on the 27 June 2006 and 7th June 2010 (reference numbers 05/Q1506/107 and 10/H1010/4 respectively). Secondary use of this data for the current study (ESMI-II: The EffectivenesS and cost effectiveness of community perinatal Mental health services study) was approved by the WCHADS data custodians and required no further ethical approvals.

### 2.3 Measures

#### 2.3.1 Observation of mother-infant interaction

Mother–child interactions were videotaped during a semi structured 15-min play session in a purpose-built room in the study base when the index child was 6–8 months old. Mothers were asked to “play as you might usually do with your baby.” Coding focused on the first 7 min of interaction, in which each dyad played with a toy of the mother’s choice from home, following the NICHD Early Child Care Research Network procedure (1999).

##### 2.3.1.1 The NICHD coding scheme

The revised manual for The Qualitative Ratings for Parent–Child Interaction for 3–15 Months of Age (Cox and Crnic, 2006) which uses 5-point global ratings adapted from the 4-point NICHD-SECYYD system (Owen, 1992), was used yielding ratings for nine maternal and four infant-focused scales and one dyadic scale. See Supplementary materials S1 for a summary description of the focus for each scale. The manual gives detailed descriptions of the characteristics of an interaction required to give a particular rating on each scale. Each dimension of the interaction is rated on a global 5-point scale, ranging from 1 (not at all characteristic) to 5 (highly characteristic), once for the entire interaction. Two summary indices were also created for the analysis which combine NICHD scales, namely a 3-item composite score (NICHD-3) and the NICHD total score (Nichd_total_). These were guided by previous empirical research using the NICHD system (NICHD-3; Mills-Koonce et al., 2007) and the manual detailing all parent and dyadic scales (Cox and Crnic, 2006). The NICHD-3 score (range 3–15) was created by summing 3 scales: global sensitivity, intrusiveness (reverse scored), and positive regard. Note here that Global sensitivity represents an overall rating of parental sensitivity to distress and non-distress episodes during play. The NICHD_total_ score was the sum scale of all 7 maternal scales plus the one dyadic scale: global sensitivity, intrusiveness (reverse scored), detachment (reverse scored), positive regard, negative regard (reverse scored), animation, stimulation, and dyadic mutuality (range 8–40). High scores indicate more optimal interaction. The internal consistency of NICHD_total_ was high at alpha = 0.85 and for NICHD-3 it was satisfactory, alpha = 0.67 (Nunnally, 1978).

##### 2.3.1.2 The PIIOS coding scheme

The Parent-Infant Interaction Observation Scale (Svanberg, 2009) manual was used to rate interactions on 13 parenting dimensions which together represent overall sensitive-responsiveness according to the authors. See Supplementary materials S1 for a summary description of the focus for each dimension. The coder rated each dimension of interaction based on descriptors given for allocation to a 3-point categorical scale, assigning a score of 0 (no concern), 2 (some concern) or 4 (significant concern) for each dimension, once for the entire interaction, with the lowest score indicating the most optimal interaction and the highest, the least optimal. Once all dimensions were assigned a score, a total score was calculated, hereafter referred to as the PIIOS_total_. The total score was then used to create the PIIOS domain score (Piios_domain_) which indicates which category the dyad’s overall quality of interaction should be allocated to; no concerns (PIIOS_total_ scores between 0 and 17), some concerns (PIIOS_total_ scores between 18 and 25) or significant concerns (PIIOS_total_ scores of 26+). The internal consistency for the PIIOS_total_ was high, alpha = 0.82 (Nunnally, 1978).

#### 2.3.2 Outcome measures

##### 2.3.2.1 Attachment security

Infant–mother attachment was assessed at 14 months of age using the Strange Situation Paradigm (Ainsworth et al., 1978). An independent trained rater from Howard Steele’s lab in the U.S. who was blind to all other study data coded all tapes and assigned them to Secure, Avoidant, Resistant or Disorganized categories. To evaluate inter-rater reliability, 53 strange situations (20%) were selected randomly for coding by a second trained blind rater. Inter-rater reliability on the four-way classification was excellent (81% exact agreement; kappa = 0.72; Landis and Koch, 1977).

Of the 250 children with dyadic videos, 234 completed the strange situation paradigm, of which three were assigned ‘cannot classify’ and were not included in analyses (total = 231). In the four-way classification, 108 (46.8%) of children were secure, 75 (32.5%) were disorganized, 26 (11.3%) were avoidant and 22 (9.5%) were resistant. For analyses, we created two binary outcome variables: secure = 0/insecure = 1 and organized = 0/disorganized = 1.

##### 2.3.2.2 Socio-emotional and behavioral development – age 1 and 2

Parents completed the Brief Infant Toddler Assessment (BITSEA; Briggs-Gowan et al., 2004) at age 1 and 2. The BITSEA is a brief 42-item screener for parents to identify children experiencing social–emotional/behavioral problems. Using a 3-point Likert scale, mothers indicated how accurate a range of statements were for their infant (0 = not true/rarely, 1 = somewhat true/sometimes, and 2 = very true/often). The BITSEA has good validity and reliability (Briggs-Gowan et al., 2004), and validity for both the internalizing and externalizing subscales has been established against diagnostic interview (Briggs-Gowan et al., 2013) in a mixed 24–48 month old sample of referred and non-referred children. We therefore focused on externalizing (6 items) and internalizing problem (8 items) subscales to index mental health outcomes in our study. Although the internal consistency of the internalizing and externalizing subscales are not reported for the standardisation sample in the BITSEA user manual or other reports by the authors (e.g., Briggs-Gowan et al., 2004), their subsequent study of referred and non-referred 2–3 year old children [mean age 36.9 months (SD 6.8)] reported internal consistency to be α = 0.80 and 0.82, respectively (Briggs-Gowan et al., 2013).

In our community sample internal consistency for externalizing problems was acceptable at both age 1 (alpha = 0.62) and age 2 (alpha = 0.72) but for internalizing the internal consistency was lower at both time points, age 1 (alpha = 0.44) and age 2 (alpha = 0.42) which may be a function of the comparatively young age of the children at each time point when problems may just be emerging, lower homogeneity in the expression of internalizing problems at this stage and the fact that this was a community sample.

For ROC analyses, prediction was examined in relation to the top 10% of total subscale scores.

#### 2.3.3 Covariates

Mother’s age was recorded at consent and child sex was recorded at birth. Socioeconomic status was derived from post code data using the English Index of Multiple Deprivation (IMD) (Noble et al., 2004) and converted to quintile categories with a binary variable (1 = most deprived, 0 = all 4 other quintiles) used for analysis.

### 2.4 Design and procedure

#### 2.4.1 Training phase

Two research assistants (psychology graduates) were trained to use the NICHD coding system by a gold standard rater. Standard training comprises 2 days plus a review session with feedback during the practice period. Raters used practice tapes to familiarize themselves with the coding scheme during training phases, seeking clarification when necessary. Practice tapes (14 observations) were drawn from the Wirral dataset and therefore not used in later analysis. Each rater then achieved inter-rater reliability for each coding dimension with a gold standard rater on another set of 30 videos from the Wirral dataset (ICCs = 0.72–0.95).

For the PIIOS coding scheme, the same raters took part in a two-day training course held by the University of Warwick. During standard training, raters completed a practice set of 14 observations before receiving feedback from the trainer that they were ready to complete a reliability set of N = 30 videos of 3-min interactions. All videos derived from either recorded health visitor home visitations or archival footage from the Sunderland Infant Programme. All raters achieved reliability (ICCs = 0.74–0.80) and then attended a further 1 day session with the trainer for feedback on results.

#### 2.4.2 Main study

Study participants’ videos were randomly allocated into blocks (*N* = 10 blocks). Eight had *N* = 26 dyads and two had 21 dyads allocated (total *n* = 250). All videos were coded using both schemes (NICHD and PIIOS). One rater was randomly allocated to independently code each block using one scheme or the other. The other rater was automatically allocated to use the alternative coding scheme for that block. This ensured no rater coded the same video using both schemes. A third rater, the gold standard rater, was randomly allocated eight videos from each block to independently code, half of which using NICHD (*n* = 40; 16%) and half using PIIOS (*n* = 40; 16%). This was to enable inter-rater reliability to be assessed for the study dataset and for each coding scheme.

An incremental approach was taken to coding each video. Each rater coded the 3, then 5, then 7-min interaction clip for the same dyad to enable examination of any incremental improvement afforded in reliability or predictive validity by increasing the observational period. Notes were written throughout the process of coding to help raters recall key aspects of the interactions that contribute to a higher or lower rating (as is normal during coding using PIIOS and NICHD). Interactions were first scored after viewing the 3-min segment of footage. This then contributed to the 5-min global rating, which then contributed to the 7-min global rating. Rating each interaction in this incremental manner using the full NICHD coding scheme took approximately 30–45 min and took 20–30 min using the full PIIOS coding scheme.

### 2.5 Statistical analysis

We evaluated the psychometric properties of the two mother-infant interaction measures based on COSMIN (Mokkink et al., 2018) and contemporary psychometric guidelines (Vitoratou et al., 2023).

#### 2.5.1 Reliability

Agreement coefficients were used to evaluate the agreement between the raters (inter-rater reliability) and subsequently agreement of ratings within rater for different observation duration (stability). Raters were Rater 1 (R_1_), Rater 2 (R_2_) and the golden criterion rater (GR). Inter-rater reliability was estimated between R_1_ vs. GR, R_2_ vs. GR, and combined ratings of R_1_ and R_2_ vs. GR. Data from all three raters were used. As the PIIOS and NICHD codes are rated in skewed three and five points scales respectively, we used the nonparametric Psi coefficient (package nopaco) (Kuiper and Hoogenboezem, 2019), which can be transformed to the intraclass correlation coefficient (ICC; Shrout and Fleiss, 1979). For completeness, we also present the percentage of agreement and Cohen’s weighted kappa coefficient where appropriate. Landis and Koch (1977) guidelines were followed for interpreting the results (values <0 no agreement; 0–0.20 slight agreement; 0.21–0.40 fair agreement; 0.41–0.60 moderate agreement; 0.61–0.80 substantial agreement; and 0.81–1 almost perfect agreement).

#### 2.5.2 Exploratory factor analysis

Exploratory factor analysis was carried out to assess the latent constructs of the NICHD and PIIOS, for ordered categorical items, using the weighted least squares mean and variance adjusted (WLSMV) estimator (Muthén et al., 1997). Measures of relative and absolute fit were used to assess the goodness of fit of the emerged structures. Latent variable analysis was conducted using Mplus software (Muthén and Muthén, 1998-2017). This data driven approach was taken to determine if coding dimensions might form factors or subscales for each measure. Any such subscales might then be examined for their predictive validity in relation to the outcomes of interest in this study, alongside those selected on an *a priori* basis from the literature or original scoring in the manual (NICHD-3, NICHD_total_, PIIOS_total_, PIIOS_domain_).

#### 2.5.3 Predictive validity

We first used logistic regression to compute the differences in the odds of receiving a (1) secure versus an insecure attachment rating and (2) a disorganized versus an organized attachment status predicted by each of the NICHD and PIIOS codes, as well as predicted by the NICHD-3, NICHD_total_ PIIOS_domain_ and PIIOS_total_ scores, for different durations of observation.

We next created receiver operating curves (ROC) (Hanley and McNeil, 1982) to test the discriminant validity of NICHD-3, NICHD_total_ PIIOS_domain_ and PIIOS_total_ in the prediction of attachment outcomes and top decile scores on each mental health outcome at different durations of observation. We further tested the discriminant validity of factor scores derived from the EFA analysis above for PIIOS and NICHD systems in relation to top decile mental health outcomes.

The ROC curve is a graphical plot of the true positive rate (sensitivity) and the false positive rate (1-specificity), where the sensitivity is the ability of a measure (e.g., NICHD-3 or PIIOS_total_) to correctly distinguish true cases (e.g., those who go on to have a top decile score on the mental health outcome) as positive. The specificity is the ability of the measure to distinguish true negatives, i.e., those without later top decile scores, as negative. Discriminative validity was evaluated by the area under the ROC curve (AUC) where values 0.9–1 indicate very good validity, 0.8–0.9 good, 0.7–0.8 fair, 0.6–0.7 poor validity and 0.5–0.6 failed to provide evidence for validity. Where the AUC indicated fair or good discriminative validity we used the Youden J (Youden, 1950) criteria, for which J = sensitivity + specificity – 1 and a perfect score is equal to one, to aid in the identification of the optimal cut-off point, given by the highest value for J. In summary, three criteria were used to determine the optimal cut-point for each scale (sensitivity, specificity and Youden’s J).

We also tested the ways in which demographic covariates might affect the ROC curves. First, we tested if a covariate (child’s sex, maternal age and level of deprivation) affects the ability of the measure to discriminate between cases and controls. Second, we tested if the ROC curve is biased by the levels of the covariate. We used the ROC regression (*rocreg*) process for the testing of significant covariates.

Finally, we used Regularized methods (Lasso Regression) and cross-validation (Machine Learning) methods to examine the prediction of attachment classification and symptom outcomes. All analyses were conducted in Stata version 16 (StataCorp, 2019) unless otherwise stated.

## 3 Results

### 3.1 Descriptives

The descriptive indices of the measuring tools (BITSEA, NICHD, and PIIOS) are presented in Supplementary Table S1.

Basic comparisons were conducted to examine whether the sample who responded to follow-up at 1 year and 2 years, were different from non-responders on the basis of parenting quality at 6–8 months or demographic characteristics and found very few differences. Those who provided data at 1 year follow-up for attachment security (*n* = 231) did not differ significantly from non-responders (*n* = 19) in terms of NICHD_total_ [t(1,248) = 0.83, *p* > 0.05], NICHD-3 [t(1,248) = 1.61, *p* > 0.05] or PIIOS_total_ scores [t(1,248) = −0.56, *p* > 0.05] at 6–8 months of age. Nor did they differ in terms of SES based on IMD [Chi Square (1) = 0.18, *p* > 0.05] or maternal age [t(1,248) = 1.80, *p* > 0.05]. We did find that a significantly higher proportion (57.9%) of those who did not complete the age 1 assessment left school before age 18 [Chi (1) = 5.05, *p* = 0.025] compared to responders (32.5%).

These findings were very similar in relation to mental health outcomes. Participants who provided data at the 1 year and 2 years follow-up for mental health outcomes (*n* = 219) did not differ significantly from non-responders (*n* = 31), in terms of NICHD_total_ [t(1,248) = 0.84, *p* > 0.05], NICHD-3 [t(1,248) = 1.17, *p* > 0.05] or PIIOS_total_ scores [t(1,248) = −0.12, *p* > 0.05]. Nor did they differ in terms of SES [Chi Square (1) = 0.86, *p* > 0.05] or maternal age [t(1,248) = −1.77, *p* > 0.05]. Again, a higher proportion (61.3%) of non-responders to the age 2 assessment left school before age 18 [Chi (1) = 11.34, *p* < 0.001] compared to responders (30.6%).

### 3.2 Inter-rater and intra-rater reliability

The interrater reliability was evaluated between two trained raters (R_1_ and R_2_) and a gold standard rater (GR). The % agreement, the Psi coefficient and the ICC per code, per observation period, is presented in Supplementary Table S2 and findings are summarized below.

#### 3.2.1 NICHD

For all NICHD individual codes, the two raters had excellent inter-rater agreement with the gold standard rater, regardless of the length of observation. The percentage of agreement across codes was at least 84%, the PSI coefficient varied between 0.72 to 0.94, leading to an ICC coefficient of at least 0.83 (excellent agreement). For 3 min of observation percentage agreement ranged from 93 to 99%, PSI ranged from 0.74–0.84, ICC was at least 0.84. For 5 min of observation percentage agreement ranged from 94 to 99%, PSI ranged from 0.75–0.88, ICC was at least 0.84. For 7 min percentage agreement ranged from 90 to 98% agreement, PSI ranged from 0.79–0.89, ICC was at least 0.85.

In terms of stability of the NICHD_total_ score, the ICC between 3 and 5 min was 0.91, the ICC between 5 and 7 was 0.97, and the ICC between the 3 and 7 min was 0.87, indicating excellent agreement in all cases. Similar results occurred for the NICHD-3, with the ICC between 3 and 5 min being 0.88, the ICC between 5 and 7 being 0.94, and the ICC between the 3 and 7 min being 0.84.

#### 3.2.2 PIIOS

For all PIIOS codes there was high inter-rater agreement between the two raters and the gold standard rater for all observation periods, across all codes. The percentage of agreement was at least 84%, the PSI coefficient varied between 0.71 to 0.96, leading to ICC coefficients of at least 0.83 (excellent agreement). For 3 min of observation percentage agreement ranged from 75 to 99%, PSI ranged from 0.75–0.90, ICC was at least 0.84. For 5 min of observation percentage agreement ranged from 89 to 99%, PSI ranged from 0.75–0.89, ICC was at least 0.84. For 7 min percentage agreement ranged from 89–99% agreement, PSI ranged from 0.75–0.93, ICC was at least 0.84.

In terms of the stability for the PIIOS_total score_, the ICC between 3 and 5 min was 0.82 and the ICC between 5 and 7 min was 0.91, while the ICC between 3 and 7 min was lower at 0.69. These results indicate that adding the first 2 min makes a small difference in the reliability scores, but adding the second 2 min does not make a difference in scores. With respect to the PIIOS_domain_ score, as expected the agreement was lower due to the categorical classification. Using weighted Kappa the agreement coefficients were 0.51 between 3 and 5 min, 0.44 between 3 and 7 min, and 0.78 between 5 and 7 min, indicating as previously that the observations at 5 and 7 min are in high agreement.

In the interest of parsimony and since 5 min observation was found to be optimal to achieve an excellent level of stability in mother-infant interaction ratings we focus next on findings for 5 min of observation for the analysis of predictive validity but highlight where results differed for shorter or longer observation periods.

### 3.3 Predictive validity

#### 3.3.1 Predicting attachment status from NICHD

In Logistic Regression the odds of secure attachment increased significantly with each one point increment in the scores of several NICHD codes for 5 min of observation (Table 2); namely 86% increment related to ‘sensitivity to non-distress’, 88% related to ‘global sensitivity’, 53% for ‘positive regard’, 53% ‘stimulation’, and 70% ‘dyadic mutuality’. A significant decrease in the odds of secure attachment by 20% is observed for each unit of increase in child ‘negative mood’ and by 32% for maternal withdrawn behavior. These effects influenced the NICHD-3 and NICHD_total_ scores, which increase the odds of secure attachment by 27 and 11% respectively, for each unit of increment. However, in ROC analyses this increase in the odds was not large enough to be translated to sufficient predictive validity for use as a screening tool, as the AUC was <0.70, for both NICHD-3 and NICHD_total_ predictors. Results for ROC analyses were very similar for all durations of observation (AUC range 0.61–0.65).

**Table 2:** Odds ratios for secure attachment and disorganized attachment predicted from NICHD scores using 5 min of observation.

NICHD predictor	Secure attachment					Disorganized attachment				
	OR	SE	p	95% CI		OR	SE	p	95% CI	
				LL	UL				LL	UL
1.Sensitivity - distress	1.10	0.05	0.029	1.0	1.2	0.93	0.04	0.101	0.8	1.0
2.Sensitivity – non-distress	1.86	0.29	<0.001	1.4	2.5	0.69	0.11	0.019	0.5	0.9
3.Global sensitivity	1.88	0.30	<0.001	1.4	2.6	0.66	0.10	0.009	0.5	0.9
4.Intrusiveness	0.78	0.11	0.081	0.6	1.0	1.32	0.19	0.057	1.0	1.7
5.Detachment	0.68	0.10	0.010	0.5	0.9	1.13	0.16	0.389	0.9	1.5
6.Positive regard	1.53	0.21	0.002	1.2	2.0	0.77	0.11	0.052	0.6	1.0
7.Negative regard	0.79	0.20	0.349	0.5	1.3	1.10	0.28	0.718	0.7	1.8
8.Animation	1.28	0.16	0.052	1.0	1.6	0.90	0.12	0.406	0.7	1.2
9.Stimulation	1.53	0.22	0.002	1.2	2.0	0.81	0.12	0.147	0.6	1.1
10.Child positive mood	1.24	0.16	0.104	1.0	1.6	0.92	0.13	0.533	0.7	1.2
11.Child negative mood	0.80	0.10	0.064	0.6	1.0	1.19	0.15	0.161	0.9	1.5
12.Child activity	1.00	0.15	0.981	0.7	1.3	1.07	0.17	0.671	0.8	1.5
13.Sustained attention	1.19	0.17	0.217	0.9	1.6	1.09	0.16	0.570	0.8	1.4
14.Dyadic mutuality	1.70	0.26	<0.001	1.3	2.3	0.73	0.11	0.036	0.5	1.0
NICHD-3	1.27	0.08	<0.001	1.1	1.4	0.84	0.05	0.006	0.7	1.0
NICHD_total_	1.11	0.03	<0.001	1.1	1.2	0.94	0.03	0.028	0.9	1.0

p, *p*-value; OR, odds ratio; SE, standard error; CI, confidence interval; LL, lower limit; UL, upper limit; NICHD, National Institute of Child Health and Human Development coding scheme.

In Logistic Regression the odds of being classified as disorganized at 1 year of age reduced significantly by 34% with one unit increase (one point) in ‘global sensitivity’ and by 27% with one unit increase in the score for ‘dyadic mutuality’ on the NICHD with 5 min of observation. For the summary scales, the odds of disorganized attachment decreased significantly by 6% for each unit increase in the NICHD-3 scale score (min score 3, max score 15) and by 6% for each unit increase in the NICHD_total_ 8 item scale (min score 8, max score 40). As in the case of secure attachment, in ROC analyses these changes in the odds are not large enough to be translated to sufficient predictive validity for the total and composite scores to be used as a screening tool, as the AUCs were only 0.62 and 0.59, respectively. Again, results were similar for all durations of observation (0.51–0.69).

#### 3.3.2 Predicting attachment status from PIIOS

In Logistic Regression the odds of secure attachment decreased with an increase of one point in the score for several PIIOS coding dimensions after 5 min of observation (Table 3). Note that higher scores on each code represent the presence of increasing concerns; there was 27% reduced odds for secure attachment in the context of poorer ‘affective engagement and synchrony’, 29% reduced odds related to poorer ‘warmth and affection’, 26% reduced odds related to poorer ‘attunement to distress’, and a 20% reduction in the context of poorer ‘responsive turn taking’. These effects influence the PIIOS_domain_ and PIIOS_total_ scores, which when increased by one unit decrease the odds of secure attachment by 35 and 3%, respectively, (the latter marginally significant; see Table 3). These translated to poor predictive validity in ROC analyses, as the AUC was only 0.56 and 0.57, respectively, for the PIIOS_domain_ and PIIOS_total_ scores. Results for ROC analyses were very similarly poor for all durations of observation (0.41–0.53).

**Table 3:** Odds ratios for secure attachment and disorganized attachment predicted from PIIOS scores using 5 min of observation.

PIIOS predictor	Secure attachment					Disorganized attachment				
	OR	SE	*p*	95% CI		OR	SE	*p*	95% CI	
				LL	UL				LL	UL
1.Infant positioning	0.88	0.1	0.288	0.7	1.1	0.97	0.12	0.782	0.8	1.2
2.Eye contact	0.95	0.09	0.565	0.8	1.1	1.03	0.1	0.749	0.9	1.2
3.Vocalizations	0.79	0.1	0.06	0.6	1	1.12	0.15	0.398	0.9	1.4
4.Affective engagement	0.73	0.1	0.018	0.6	0.9	1.02	0.14	0.864	0.8	1.3
5.Warmth	0.71	0.09	0.009	0.5	0.9	1.08	0.15	0.562	0.8	1.4
6.Holding /handling	0.87	0.09	0.191	0.7	1.1	1	0.12	0.972	0.8	1.3
7.Verbal commenting	0.95	0.12	0.663	0.7	1.2	0.99	0.13	0.91	0.8	1.3
8.Attunement to distress	0.74	0.1	0.022	0.6	1	1.15	0.15	0.295	0.9	1.5
9.Intrusiveness	1.08	0.13	0.517	0.9	1.4	0.98	0.13	0.898	0.8	1.3
10.Expectations	0.92	0.11	0.489	0.7	1.2	1.16	0.14	0.24	0.9	1.5
11.Empathic	0.89	0.11	0.347	0.7	1.1	1.1	0.14	0.447	0.9	1.4
12.Responsive	0.8	0.1	0.056	0.6	1	1.17	0.15	0.221	0.9	1.5
13.Infant self-soothing	1.14	0.16	0.334	0.9	1.5	0.82	0.12	0.18	0.6	1.1
PIIOS_domain_	0.65	0.14	0.047	0.4	0.9	0.99	0.02	0.545	1	1
PIIOS_total_	0.97	0.02	0.051	0.9	1	1.08	0.24	0.727	0.7	1.7

p, *p*-value; OR, odds ratio; SE, standard error; CI, confidence interval; LL, lower limit; UL, upper limit; PIIOS, parent-infant interaction observation scale.

In Logistic Regression the odds of disorganized attachment did not alter based on PIIOS item, domain, or total scores (Table 3). The area under the curve additionally indicated poor or failed predictive validity (AUC 0.53 for the PIIOS_total_ score and 0.51 for the PIIOS_domain_ score). Results for ROC analyses were similarly poor for all durations of observation (0.46–0.54).

#### 3.3.3 Predicting mental health outcomes at age 1 and 2 from NICHD

The ROC analysis revealed that NICHD-3 ratings made from 5 min observation show fair predictive validity to ‘externalizing’ problems at age 1 and 2 with AUC = 0.74 and 0.74, respectively, (Tables 4, 5) shows the optimal cut-off point, as suggested by the sensitivity and specificity, for NICHD-3 where area under the curve was ≥0.70 for prediction to age 2 top decile externalizing problems. A score of 11 or below on the NICHD-3 had 92.8% sensitivity and 52.2% specificity to detect membership of the top decile at age 1, correctly classifying 54%. Whereas a score of 10 or below had 72.7% sensitivity and 66.3% specificity to detect membership of the top decile for externalizing problems at age 2, with correct classification of 67%. The values for AUC were very similar for 7 min of observation but sub-threshold for 3 min observation (See Supplementary Table S3). For 5 min observation NICHD_total_ only predicted externalizing (AUC > 0.70) at age 1 but not at age 2 which suggests that use of the brief NICHD-3 is optimal for the NICHD-SECCYD system. Neither NICHD index predicted internalizing problems at the threshold required.

**Table 4:** Area under the curve analysis results for prediction of child mental health at age 1 and 2 based on NICHD (5 min observation).

	BITSEA	Unadjusted			Adjusted			Covariates
		AUC	95% CI		AUC	95% CI		
			LL	UL		LL	UL	
NICHD-3	Externalizing (age 1)	**0.74**	0.64	0.83	**0.71**	0.62	0.80	Maternal age
	Internalizing (age 1)	0.48	0.27	0.68	0.47	0.26	0.68	Maternal age
	Externalizing (age 2)	**0.74**	0.54	0.89	0.66	0.49	0.84	Maternal age
	Internalizing (age 2)	0.58	0.35	0.80	0.54	0.31	0.78	Maternal age
NICHD total score	Externalizing (age 1)	0.69	0.58	0.80	0.66	0.55	0.77	Maternal age
	Internalizing (age 1)	0.51	0.29	0.73	0.50	0.28	0.72	Maternal age
	Externalizing (age 2)	0.66	0.48	0.84	0.61	0.42	0.80	Maternal age
	Internalizing (age 2)	0.49	0.26	0.73	0.47	0.21	0.73	Maternal age

AUC, area under the curve; CI, confidence interval; LL, lower limit; UL, upper limit; NICHD, National Institute of Child Health and Human Development coding scheme; BITSEA, brief infant toddler social–emotional scale. Values for AUC above the threshold of ≥0.70 are shown in bold.

**Table 5:** The optimal cut-off points, as suggested by the sensitivity and specificity, for the NICHD-3 composite score in the prediction of externalizing problems at age 1 and 2 (AUCs ≥0.70).

	BITSEA	Cut point	Sensitivity (%)	Specificity (%)	Youden J
NICHD-3 composite score	Externalizing (age 1)	≤13	100.00	24.39	0.244
		≤12	100.00	38.05	0.381
		≤11	92.86	51.22	0.441
		≤10	64.29	65.85	0.301
		≤9	50.00	80.00	0.300
	Externalizing (age 2)	≤12	90.91	37.98	0.289
		≤11	81.82	52.40	0.342
		≤10	72.73	66.35	0.391
		≤9	36.36	77.88	0.142
		≤8	36.36	91.83	0.282

NICHD, National Institute of Child Health and Human Development coding scheme; BITSEA, brief infant toddler social–emotional scale.

#### 3.3.4 Predicting mental health outcomes at age 1 and 2 from PIIOS

According to the ROC analysis, the PIIOS_total_ from 5 min of observation had fair to good predictive validity to ‘externalizing’ (AUC = 0.82) and ‘internalizing’ (AUC = 0.72) BITSEA subscales at age 2 (see Table 6). Predictive validity from PIIOS_domain_ was fair for externalizing but sub-threshold for internalizing. Table 7 shows the optimal cut-off points in relation to sensitivity and specificity for the PIIOS_total_ in the prediction of child mental health outcomes at age 2, where AUC was ≥0.70. A score of 20 or above had 81.8% sensitivity and 74.5% specificity to detect membership of the top decile for externalizing problems, with 75% correctly classified, and had 66.7% sensitivity and 73.3% specificity (73% correctly classified) to detect membership of the top decile for internalizing problems at age 2.

**Table 6:** Area under the curve analysis results for BITSEA prediction based on PIIOS (5 min observation).

	BITSEA	Unadjusted			Adjusted			Covariates
		AUC	95% CI		AUC	95% CI		
			LL	UL		LL	UL	
PIIOS domain score	Externalizing (age 1)	0.61	0.45	0.76	0.51	0.30	0.72	Child sex; deprivation
	Internalizing (age 1)	0.58	0.42	0.73	–			–
	Externalizing (age 2)	0.77	0.64	0.91	0.67	0.49	0.85	Child sex
	Internalizing (age 2)	0.69	0.51	0.86	0.61	0.35	0.86	Child sex; maternal age
PIIOS total score	Externalizing (age 1)	0.66	0.51	0.81	0.63	0.47	0.79	Child sex; deprivation
	Internalizing (age 1)	0.58	0.42	0.74	0.55	0.40	0.70	Child sex; deprivation
	Externalizing (age 2)	0.82	0.72	0.93	0.78	0.67	0.90	Child sex
	Internalizing (age 2)	0.72	0.55	0.90	0.70	0.50	0.90	Child sex; maternal age

AUC, area under the curve; CI, confidence interval; LL, lower limit; UL, upper limit; BITSEA, brief infant toddler social–emotional scale; PIIOS, parent-infant interaction observation scale.

**Table 7:** The optimal cut-off points, as suggested by the sensitivity and specificity, for the PIIOS total score in the prediction of externalizing and internalizing problems at age 2 (AUCs ≥0.70).

	BITSEA	Cut point	Sensitivity (%)	Specificity (%)	Youden J
PIIOS total score	Externalizing (age 2)	≥ 24	81.82	60.10	0.419
		≥ 22	81.82	69.23	0.511
		≥ 20	81.82	74.52	0.563
		≥ 18	72.73	80.77	0.535
		≥ 16	45.45	88.46	0.339
	Internalizing (age 2)	≥ 24	66.67	59.05	0.257
		≥ 22	66.67	68.10	0.348
		≥ 20	66.67	73.33	0.400
		≥ 18	55.56	79.52	0.351
		≥ 16	33.33	87.62	0.210

PIIOS, parent-infant interaction observation scale; BITSEA, brief infant toddler social–emotional scale.

The values for AUC from PIIOS_total_ were very similar for 5 (0.78) and 7 min (0.77) of observation for externalizing problems, but for 3 min AUC fell below the <0.70 threshold in the prediction of Internalizing problems at age 2 (0.68; Supplementary Table S4). All AUCs for PIIOS_total_ and PIIOS_domain_ scores fell well below threshold at <0.70 for prediction to age 1 outcomes. These findings indicate that use of PIIOS_total,_ rather than the domain based scoring system, is optimal for this tool.

#### 3.3.5 Effect of covariates on mental health outcome prediction

In ROC regression analyses, ROC curves at specific values of the covariates were not implemented, as no covariates were identified as having a significant effect on the discriminatory ability of the NICHD-3 or NICHD_total_ or PIIOS_total_ and PIIOS_domain_ scores. When adjusting the ROC curves for the presence of covariates, maternal age was found to have a significant effect on the performance of NICHD-3 and NICHD_total_ score for both BITSEA outcomes variables. For the PIIOS the three covariates, namely the sex of the child, maternal age and deprivation, were each found to have a significant effect on performance. The trend in all cases was a marginal fall in the value of AUC for the covariate-adjusted ROC curve with covariates. Tables 4, 6 show the differences between the adjusted and unadjusted ROC curves.

### 3.4 Further exploratory analysis

We conducted exploratory analyses to determine if alternative, potentially optimal summary scores could be extracted from PIIOS and NICHD, either simplified into overall scores using Factor Analysis or representing combinations of codes and interactions between codes derived from machine learning techniques.

Factor analysis for categorical data was performed for PIIOS and NICHD systems separately to establish whether or not a new combination of parenting codes might prove reliable and predict later outcomes. Factor Analysis led to a 2-factor model in each case. The factors derived for each measure were of good or satisfactory internal consistency and content validity. However, when ROC analyses were conducted to assess predictive validity to attachment and mental health outcomes, Factor Scores had poor discriminant validity in the case of prediction to each mental health outcome at age 1 and 2 and attachment outcome at age 1 (AUC < 0.70). We concluded that the predictive validity was not increased if the information from NICHD or PIIOS was summarized using a different clustering of the codes, to that proposed *a priori* in the Measures section above, for either tool. See Supplementary materials S2 for details of these analyses.

Regularized methods (Lasso regression) and cross-validation (Machine Learning) were used to examine the prediction of attachment classification and mental health symptom outcomes. Both the NICHD and PIIOS coding schemes provide ratings of a range of behaviors, some principally of the mother, some of the child and some that are intrinsically dyadic in nature. We considered the possibility that there might be coding dimensions and combinations of those dimensions within each measure, that might be of salience for later development of secure attachment and behavior problems. Analyses revealed that the *a-priori* assumed scores almost always performed better than any other combination of codes, for both PIIOS and NICHD. The results of these analyses are given in Supplementary materials S3.

### 3.5 Agreement between NICHD and PIIOS in the identification of vulnerable dyads

In our high psych-social risk community sample, the simple bivariate association between NICHD-3 and PIIOS_total_ scores was only moderate, rho = 0.41. Using the cut points for sensitivity and specificity derived above identified 28.3% of dyads as at risk according to PIIOS_total_ and 24% dyads as high risk on NICHD-3. Although the overall agreement level in classification of dyads as high or low risk was 72.4%, so a dyad that was rated high risk on PIIOS was 4 times more likely to be rated as high risk on NICHD (OR = 4.0, 95% CI: 2.2, 7.4, *p* < 0.001), the two scales also appeared to detect slightly different forms of risk.

Logistic regression revealed that those dyads identified using NICHD-3 were at raised risk of insecure attachment (OR = 2.7, 95% CI: 1.4, 5.2, *p* = 0.004) and top decile level externalizing problems at age 2 (OR = 1.9, 95% CI: 0.8, 4.6, *p* = 0.14) which suggests the threshold for externalizing problems derived above can be used to identify dyads at risk for attachment insecurity. In contrast, dyads identified using PIIOS were at raised risk for externalizing (OR = 3.8, 95% CI: 1.6, 8.7, *p* = 0.002) and internalizing problems at age 2 (OR = 2.2, 95% CI: 0.9, 5.0, *p* = 0.073) but not as clearly for insecure attachment (OR = 1.5, 95% CI: 0.9, 2.7 *p* = 0.16, ns). However, these results need to be considered with caution. Odds ratios are best used as the index of effect size here, since the small sample size constrained our power to detecting moderate effects as significant.

We calculated the comparative likelihood of being classified as insecure based on above/below threshold status on PIIOS_total_ and NICHD-3. The Odds of being insecure were threefold higher for dyads scoring above threshold on both tools (OR 3.3, 95% CI: 1.3, 8.3, *p* = 0.01), compared to dyads scoring as low risk on both measures (Overall, Chi Squared (3) = 9.08, *p* = 0.028). However, the raised risk for attachment insecurity is driven by the NICHD-3 rating as the risk of insecure attachment was not raised in those dyads who only score high on the PIIOS (OR 1.1, 95% CI: 0.5, 2.3, *p* = 0.78) but was raised for those who only score high on NICHD (OR 2.2, 95% CI: 0.9, 5.5, *p* = 0.088).

## 4 Discussion

In this study we aimed to evaluate two observational measures (NICHD and PIIOS), used to assess the quality of mother-infant interaction to inform their use in clinical services. We evaluated them in terms of their reliability, and predictive validity to age 1 attachment status and age 1 and age 2 child mental health outcomes.

Regarding raters’ reliability, two naïve raters were trained on both measures and each was able to achieve an excellent level of reliability against a gold-standard rater on both measures when rating 3, 5, and 7 min of observation. There was evidence to suggest that agreement between ratings for 5 and 7 min was more stable than the one gained between 3 and 7 min, suggesting that observation for 5 min was optimal in terms of reliability. We therefore recommend filming for 5 min in clinical practice, a finding that was also supported by the results of validity testing discussed next.

In terms of predictive validity, we aimed to determine whether brief forms of each observation tool might possess sufficient predictive validity for use in routine outcome measurement. Our findings established that the PIIOS needs to be used in its full form (i.e., 13 items) and that the PIIOS_total_ was optimal with predictive validity to both internalizing and externalizing outcomes at age 2, superior to PIIOS_domain_. Instead, the optimal form of the NICHD coding system was the brief 3-item composite measure (NICHD-3); this composite score includes global sensitivity, positive regard (warmth) and intrusiveness (reverse scored), and it was used previously in the literature as an index of maternal sensitivity (e.g., Mills-Koonce et al., 2007). In fact, NICHD-3 predicted externalizing problems at age 1 and age 2, whereas NICHD_total_ (across 8 codes) predicted age 1 outcome only in ROC analyses. Reassuringly, our factor analytic, lasso regression and machine learning approaches to analysis of each tool confirmed there were no other combinations of codes, including interactions between codes, in either measure that were any better than these *a priori* selected approaches to combining codes, in terms of predictive validity to the outcomes under test.

ROC analyses confirmed that both the PIIOS_total_ and the NICHD-3 from 5 min of observation could be used to identify at-risk dyads with fair to good predictive validity to age 2 mental health outcomes. Scores above threshold on the PIIOS_total_ correctly classified 75% of cases and the scores above threshold on NICHD-3 correctly classified 67% of cases, in relation to later age 2 externalizing problems. On the one hand, this is impressive since in our arguably conservative approach to analysis we were using one construct (parenting) to longitudinally predict a different outcome (child mental health), rather than the conventional use of ROC to evaluate the performance of a screening tool against a diagnostic measure of the same construct often cross-sectionally. In the former case, one might expect the results for AUC to be attenuated compared to the latter. On the other hand, using other indicators of risk in addition to parent-infant interaction quality, such as presence of parental mental health problems, may further improve the accurate identification of dyads who warrant early intervention. In our study, we also showed that the validity attained from 7 min of observation was very similar to that attained from 5 min, whereas for both measures prediction was subthreshold (<=0.70) from 3 min of observation. This finding also supports the use of a five-minute observation period in clinical practice. Although there is no previous published work on the predictive validity of PIIOS, previous work using NICHD in research settings supports the findings that the three-item composite predicts later adverse child outcomes, but longer observation periods have typically been used. Our findings are in line with previous work summarized in a meta-analytic review confirming direct associations between observed maternal sensitivity in the first year of life and childhood emotional and behavior problems (Cooke et al., 2022), and they extend these findings by confirming predictive validity can be achieved with only 5 min of observation.

Neither measure predicted attachment security or disorganization at the high-bar level required for a screening tool in our study, with AUC > 0.70. However, this is in line with published meta-analyses where a combined effect size of around 0.24 from maternal sensitivity to attachment security assessed using the Strange Situation Paradigm has been found in community samples, suggesting that the magnitude of prospective association is typically small to moderate (e.g., De Wolff and Van Ijzendoorn, 1997). Our findings from logistic regression are also concordant with this. We found several parenting dimensions in each measure that were significantly associated with later attachment security. Each unit increase in the NICHD-3 composite score (range 0–15; high scores represent optimal parenting) was associated with a 27% increase in likelihood of secure attachment at age 14 months. Whereas each unit decrease in the PIIOS_total_ score (range 0–40; low scores represent optimal parenting) was associated with a 3% increased likelihood of secure attachment. In addition, each unit increase in the NICHD-3 composite score was associated with a 16% decrease in likelihood of disorganized attachment at age 14 months. However, none of the parenting dimensions assessed by PIIOS were significant in the prediction of attachment disorganization.

The PIIOS was developed to identify at-risk dyads for insecure attachment and it was originally validated against the maternal sensitivity scale of Infant CARE Index (Crittenden, 2001). Yet our findings from logistic regression provide only weak support for the prediction to attachment security. Certainly, since no other studies have reported on prospective prediction to attachment outcomes from PIIOS, our findings require replication. However, the fact that infants who were identified as at-risk for later externalizing problems using the NICHD-3 index at 6–8 months of age were also found to be nearly three times more likely to be insecure at 14 months (OR 2.7) suggests NICHD-3 may be identifying vulnerability to attachment security, as assessed by the Strange Situation, more effectively than the PIIOS since infants at-risk for externalizing problems on PIIOS were only found to be at slightly increased risk (OR 1.5). Future studies might usefully determine whether PIIOS is more effective at identifying dyads who go on to be deemed at-risk using Crittenden’s Toddler CARE Index.

### 4.1 Overlap in identifying dyads at risk of internalizing and externalizing problems

Based on NICHD-3 22.8% of dyads were deemed at risk for externalizing problems at age 2 with scores of 10 or below. Based on PIIOS_total_ scores 28.3% were deemed at risk for internalizing and/or externalizing problems with scores of 20 or above. Overall, there was 72.4% agreement in the identification of dyads as at-risk or not, however cross-tabulation of risk status revealed that 16% of dyads were identified as at-risk on PIIOS_total_ only and 11.6% on NICHD-3 only. This may have arisen since PIIOS more effectively identified dyads in which the infant is vulnerable to developing internalizing problems whereas the NICHD-3 was more effective at identifying dyads vulnerable to insecure attachment. A clue as to why this might be lies in the fact that the theoretical origins and approach to dyadic coding within the two measures differs. PIIOS, like Crittenden’s CARE Index, uses more explicit observation of the dyadic-interplay in the play context, whereas the NICHD-SECCYD system codes maternal and infant behavior separately, albeit from the interaction. Also, the *a priori* selected summary scores from the NICHD system, the NICHD_total_ and NICHD-3, focused on parental behavior scales only. The PIIOS may therefore more explicitly assess what the infant brings to the interaction, possibly indexing infant temperament-related contributions more directly, and thus additional vulnerability to mental health problems. For example, meta-analytic findings from the broader developmental literature support the premise that infant negative emotionality (irritability) is itself associated with later internalizing and externalizing problems (Finlay-Jones et al., 2023). By contrast, NICHD-3 assessment focuses more on coding maternal behaviors, albeit in relation to their infant’s signals in the case of global sensitivity. The maternal sensitivity scale within the NICHD system is closest to that originally devised by Ainsworth to assess sensitivity (Mesman and Emmen, 2013) which was developed to predict attachment security.

### 4.2 Translation of work to clinical practice

Unlike NICHD-3, the PIIOS_total_ evidenced predictive validity to both internalizing and externalizing outcomes at age 2. As stated above, for many codes within PIIOS the rater considers the parental behavior *and* the infant’s response, which means that the developmental descriptions in the manual necessarily reflect infant capabilities at age 2–8 months of age, the time period for which the tool was designed. Whilst a strength in terms of prediction, this is also a limitation for its broader use in specialist perinatal mental health services with a wider remit to assess parenting for 0–2 years and use in other services that may serve a broader age cohort of infants. By contrast, NICHD-3 scales were developed for application up to age 2 (Mesman and Emmen, 2013) with only the nature of the play-based task changing to ensure age-appropriateness. Training for the full NICHD-SECCYD and the full PIIOS scales takes a similar amount of time (3 days face to face), but, for the brief NICHD-3, time to train would reduce to 2 days face to face. Time to become reliable and to code in routine clinical practice would also be substantially reduced, with associated cost savings, thereby enhancing its suitability as a routine outcome measure. We estimate coding the NICHD-3 scales for 5 min observation in clinical practice would take approximately 15 min, which contrasts with 35 min for the full NICHD system and 20–25 min for the PIIOS system. Finally, our prediction to age 2 mental health outcomes can be set in the context of other work confirming that BITSEA scores at mean age 23 months are clinically meaningful as they predict later adverse mental health outcomes, for instance at age 6 (Briggs-Gowan and Carter, 2008). Indeed, in the WCHADS study, from where the current dataset was drawn, BITSEA externalizing problems at age 2 shows moderate simple associations with age 9 (rho = 0.41) and age 11 (rho = 0.39) parent reports of externalizing problems on the Child Behavior Checklist and adolescent self-report of depression on the Moods and Feelings Questionnaire, rho =0.23 (personal communication) supporting the predictive validity of the clinical outcome measures in this study.

## 5 Strengths and limitations

One of the main strengths of this investigation is the use of rare, longitudinal data from a relatively large high psychosocial risk community sample, with filmed observational archive data and longitudinal follow-up to gold standard methods to assess children’s outcomes such as the Strange Situation. We ensured that independent raters of parenting were blind to attachment status and child mental health outcome and we tested the psychometric properties of two observational schemes, selected for different reasons, alongside one another. We followed contemporary best practice guidance to evaluate the psychometric properties of each tool including examination of confounder effects on the predictive validity of each scale in which we were able to show that key demographic factors did not significantly reduce the measures’ discriminative validity. Finally, we also used a novel approach to testing incremental validity over three different short observation periods in order to make recommendations for routine clinical practice.

In terms of limitations, whilst we established predictive validity for both tools to BITSEA externalizing outcomes at age 2, the cut point selected was arbitrary, set at the top tenth centile of scores, albeit within a high psychosocial-risk community sample. Future work will need to assess prediction to diagnostic outcomes and assess performance of the scales in clinical samples. In relation to internalizing problems at age 2, only PIIOS showed acceptable predictive validity. The internal consistency of the BITSEA internalizing subscale was low in our study and markedly lower that that reported in a slightly older mixed referred/non-referred sample previously (mean age 36.9 months; Briggs-Gowan et al., 2013). The items within this BITSEA subscale assess a broad range of internalizing problems (e.g., distress on separation, sadness, social withdrawal) which may emerge at different stages in the early years, may not necessarily co-occur consistently and may be harder to recognize and endorse by parents. Though, the use of a top decile cut-point as the outcome in the study is still likely to have identified a group of children with high levels of internalizing problems of a varying nature. Also, whilst the WCHADS sample included a mix of families living in mostly deprived neighborhoods but also some affluent areas, the sample has very little ethnic variation which is characteristic of the local population from which it was drawn. PIIOS was originally validated against the Care Index cross-sectionally in a socio-economically deprived sample from the Northeast of England. In contrast, the NICHD-SECCYD system was developed in US and has been used in many studies with more ethnically diverse populations. Replication of the current work is therefore required with more ethnically diverse populations. The WCHADS study filmed interactions between the main caregiver and the infant and for 100% of families this was the mother figure at 6–8 months of age, as a consequence our findings cannot be assumed to extend more broadly to other parental figures or alternative caregivers. Whilst we showed that the discriminative validity of each tool was only marginally affected by maternal demographic covariates in this study, future larger scale studies might consider accounting for other infant-related covariates which may contribute to mental health outcomes such as gestational age at birth (Xia et al., 2021).

Finally, assessing the contribution of parental sensitivity specifically to distress within interactions, which is part of the NICHD full version, was limited since during the standard NICHD-SECCYD assessment 66.3% did not show distress in the play-based task during 3 min observation, although this reduced to 57.5% during 5 min and 48.4% during 7 min of play. In anticipation of this we selected the Global sensitivity code which encompasses distress and non-distress episodes during play for inclusion in NICHD-3 following some previous studies (Mills-Koonce et al., 2007). We used a play-based observation since both the PIIOS and NICHD-SECCYD systems adopt this approach. We also felt that a play-based assessment would be more normative for use in clinical practice and would be most acceptable to women, in contrast to using distress-eliciting tasks sometimes reported in the literature. However, since previous work has shown that sensitivity to distress may be important in the prediction of attachment security (McElwain and Booth-LaForce, 2006) future studies might usefully contrast the predictive valdity of the measures when applied to play interactions with that achieved when filming mildly stressful caregiving tasks which might elicit more naturally occuring distress in young infants and enable coding this element of interactions in a higher proportion of dyads.

### 5.1 Future work

Future work should aim to replicate these findings in more ethnically diverse populations. Since early parenting is known to impact child cognitive or language outcomes, as well as developmental delay, future work should also aim to evaluate a broader spectrum of child outcomes. Whilst the NICHD-SECCYD scale has been shown to be sensitive to change in maternal sensitivity following parenting intervention (Ravn et al., 2011) evidence for both short form NICHD-3 and the PIIOS’s ability to show sensitivity to change pre-to-post intervention needs to be established for use as a routine outcome measure. Finally, although observational measures are widely agreed to be a gold standard approach providing an objective assessment of parenting, and generate different information to parental self-report which can be biased by the respondent’s mood or concerns about social-acceptability (Corcoran and Fischer, 2013), the need to film with a camera might not be acceptable to all parents accessing clinical services. Furthermore, post-assessment coding carries an additional work-load for clinicians. Future research might usefully try to establish whether reliability in the use of these validated scales can be achieved ‘live’ in session which would remove the need for post-assessment rating and might prove to be most acceptable to those being observed. At the present time we are not aware of a substantive evidence base for any ‘live’ coding systems.

## 6 Conclusion

This study evaluated two observational systems and confirmed that the PIIOS and a short form of the NICHD-SECCYD system (NICHD-3) are reliable and valid tools for identifying parent-infant interaction qualities that predict later toddler mental health outcomes. These tools identified overlapping and different forms of vulnerability in mother-infant dyads. Choice of which measure clinicians might use will depend on the likely goals for intervention, the age of the infant and time constraints on assessment within busy clinical practice. This paper may serve as a valuable resource for researchers and clinicians in both perinatal and infant mental health fields, particularly those involved in the observational assessment of mother–infant relationship quality.

## Data availability statement

The datasets presented in this article are not readily available due to ethical constraints. Supporting data are available to bona fide researchers on approval of an application for access. Further information about the data and conditions for access are available at the University of Liverpool Research Data Catalogue: doi: 10.17638/datacat.liverpool.ac.uk/564. Requests to access the datasets should be directed to HS, hmsharp@liverpool.ac.uk.

## Ethics statement

The study, which involved human participants, was approved by the Cheshire North and West Research Ethics Committee on the 27 June 2006 and on the 7th June 2010 (reference numbers 05/Q1506/107 and 10/H1010/4 respectively). The study was conducted in accordance with the local legislation and institutional requirements. Written informed consent for participation in this study was provided by the participants and by the participants’ legal guardians/next of kin.

## Author contributions

HS: Conceptualization, Funding acquisition, Investigation, Methodology, Supervision, Writing – original draft, Writing – review & editing, Project administration. SV: Formal analysis, Funding acquisition, Methodology, Writing – original draft, Writing – review & editing, Conceptualization, Investigation, Supervision. HO’M: Conceptualization, Funding acquisition, Methodology, Writing – original draft, Writing – review & editing. LB: Data curation, Investigation, Methodology, Project administration, Writing – original draft, Writing – review & editing. MR: Investigation, Methodology, Project administration, Writing – review & editing. CH: Formal analysis, Investigation, Project administration, Writing – original draft, Writing – review & editing. JG: Investigation, Methodology, Project administration, Writing – review & editing. AP: Conceptualization, Formal analysis, Funding acquisition, Investigation, Methodology, Supervision, Writing – original draft, Writing – review & editing.
