# Supplementary material for: Identifying vulnerable mother-infant dyads: a psychometric evaluation of two observational coding systems using varying interaction periods
Source: Front Psychol. 2024 Jun 24;15:1399841. doi: 10.3389/fpsyg.2024.1399841 (PMC11233099; doi:10.3389/fpsyg.2024.1399841)
Supplement: Supplementary file 1 [file Table_1.DOCX]

Supplementary Material

**Table S1.** Descriptive Indices of Measures

| Outcome variable | n | M | | SD | | | Median | | Range | |
| --- | --- | --- | --- | --- | --- | --- | --- | --- | --- | --- |
| BITSEA Ext – Age 1 | 219 | 1.8 | | 1.7 | | | 1 | | 0-11 | |
| BITSEA Int – Age 1 | 219 | 1.3 | | 1.2 | | | 1 | | 0-6 | |
| BITSEA Ext – Age 2 | 219 | 2.0 | | 1.9 | | | 1 | | 0-10 | |
| BITSEA Int – Age 2 | 219 | 1.7 | | 1.5 | | | 2 | | 0-9 | |
| Predictor variable |  |  | |  | | |  | |  | |
| NICHD _Total_ (3 mins) | 250 | 30.1 | | 4.8 | | | 31 | | 16-39 | |
| NICHD _Total_ (5 mins) | 250 | 30.1 | | 5.2 | | | 30 | | 17-40 | |
| NICHD _Total_ (7 mins) | 250 | 29.9 | | 5.4 | | | 30 | | 16-40 | |
| NICHD-3 (3 mins) | 250 | 11.4 | | 2.1 | | | 12 | | 6 - 15 | |
| NICHD-3 (5 mins) | 250 | 11.4 | | 2.2 | | | 11 | | 5 - 15 | |
| NICHD-3 (7 mins) | 250 | 11.3 | | 2.4 | | | 11 | | 5-15 | |
| PIIOS _Total_ (3 mins) | 250 | 11.5 | | 7.6 | | | 10 | | 0-38 | |
| PIIOS _Total_ (5 mins) | 250 | 13.3 | | 8.1 | | | 12 | | 0-40 | |
| PIIOS _Total_ (7 mins) | 250 | 14.2 | | 8.7 | | | 14 | | 0-38 | |
|  |  | No | | | Some | | | Sign. | | |
|  |  | n | % | | n | % | | n | | % |
| PIIOS _Domain_ (3 mins) | 250 | 189 | 75.6 | | 49 | 19.6 | | 12 | | 4.8 |
| PIIOS _Domain_ (5 mins) | 250 | 166 | 66.4 | | 66 | 26.4 | | 18 | | 7.2 |
| PIIOS _Domain_ (7 mins) | 250 | 157 | 62.8 | | 66 | 26.4 | | 27 | | 10.8 |
| Note. *NICHD* = National Institute of Child Health and Human Development coding scheme; *PIIOS* = Parent -Infant Interaction Observation Scale; *BITSEA* = Brief Infant Toddler Social-Emotional Scale; *PIIOS* *domains* = No concern, some concern, significant concern; *Int* = Internalizing problems; *Ext* = Externalizing problems. | | | | | | | | | | |
