# Supplementary material for: Identifying vulnerable mother-infant dyads: a psychometric evaluation of two observational coding systems using varying interaction periods
Source: Front Psychol. 2024 Jun 24;15:1399841. doi: 10.3389/fpsyg.2024.1399841 (PMC11233099; doi:10.3389/fpsyg.2024.1399841)
Supplement: Supplementary file 2 [file Table_2.DOCX]

**Table S2.** Range of Coefficients of Agreement over all NICHD and PIIOS Codes Between Raters (Interrater Reliability) and Durations of Observations (Stability), Across all Items and Observation Times

|  |  | NICHD | | | PIIOS | | |
| --- | --- | --- | --- | --- | --- | --- | --- |
|  |  | % Agreement | Psi | ICC | % Agreement | Psi | ICC |
| Gold | 3 min vs 5 min | 93% to 98% | 0.74 to 0.87 | 0.84 to 0.89 | 80% to 98% | 0.73 to 0.87 | 0.83 to 0.89 |
|  | 5 min vs 7 min | 97% to 99% | 0.82 to 0.90 | 0.86 to 0.90 | 95% to 99% | 0.77 to 0.90 | 0.85 to 0.90 |
|  | 3 min vs 7 min | 89% to 98% | 0.73 to 0.86 | 0.83 to 0.88 | 91% to 97% | 0.73 to 0.84 | 0.83 to 0.87 |
| Rater 1 | Rater vs Gold at 3 min | 88% to 99% | 0.72 to 0.85 | 0.83 to 0.87 | 60% to 99% | 0.96 to 0.96 | 0.84 to 0.94 |
|  | Rater vs Gold at 5 min | 84% to 99% | 0.73 to 0.90 | 0.83 to 0.90 | 90% to 98% | 0.77 to 0.95 | 0.84 to 0.93 |
|  | Rater vs Gold at 7 min | 86% to 98% | 0.75 to 0.90 | 0.84 to 0.90 | 80% to 99% | 0.75 to 0.94 | 0.84 to 0.92 |
|  | 3 min vs 5 min | 94% to 99% | 0.75 to 0.87 | 0.84 to 0.89 | 95% to 99% | 0.74 to 0.87 | 0.84 to 0.88 |
|  | 5 min vs 7 min | 97% to 100% | 0.78 to 0.89 | 0.85 to 0.89 | 96% to 100% | 0.77 to 0.91 | 0.85 to 0.90 |
|  | 3 min vs 7 min | 93% to 99% | 0.74 to 0.87 | 0.84 to 0.88 | 91% to 97% | 0.73 to 0.83 | 0.83 to 0.87 |
| Rater 2 | Rater vs Gold at 3 min | 92% to 99% | 0.75 to 0.90 | 0.84 to 0.90 | 75% to 100% | 0.71 to 0.84 | 0.83 to 0.87 |
|  | Rater vs Gold at 5 min | 94% to 100% | 0.76 to 0.91 | 0.84 to 0.90 | 85% to 100% | 0.72 to 0.84 | 0.83 to 0.87 |
|  | Rater vs Gold at 7 min | 94% to 99% | 0.81 to 0.94 | 0.86 to 0.92 | 89% to 99% | 0.73 to 0.91 | 0.83 to 0.90 |
|  | 3 min vs 5 min | 96% to 99% | 0.73 to 0.87 | 0.83 to 0.89 | 90% to 97% | 0.75 to 0.87 | 0.84 to 0.89 |
|  | 5 min vs 7 min | 97% to 99% | 0.75 to 0.90 | 0.84 to 0.90 | 95% to 99% | 0.79 to 0.93 | 0.85 to 0.91 |
|  | 3 min vs 7 min | 93% to 98% | 0.73 to 0.85 | 0.83 to 0.88 | 91% to 96% | 0.73 to 0.85 | 0.83 to 0.88 |
| Combined | Raters vs Gold at 3 min | 93% to 99% | 0.74 to 0.84 | 0.84 to 0.87 | 75% to 99% | 0.75 to 0.90 | 0.84 to 0.90 |
|  | Raters vs Gold at 5 min | 94% to 99% | 0.75 to 0.88 | 0.84 to 0.89 | 89% to 99% | 0.75 to 0.89 | 0.84 to 0.90 |
|  | Raters vs Gold at 7 min | 90% to 98% | 0.79 to 0.89 | 0.85 to 0.90 | 89% to 99% | 0.75 to 0.93 | 0.84 to 0.91 |
|  | 3 min vs 5 min | 96% to 99% | 0.74 to 0.87 | 0.84 to 0.88 | 95% to 98% | 0.75 to 0.88 | 0.84 to 0.89 |
|  | 5 min vs 7 min | 97% to 99% | 0.76 to 0.89 | 0.84 to 0.89 | 96% to 99% | 0.79 to 0.92 | 0.85 to 0.91 |
|  | 3 min vs 7 min | 93% to 99% | 0.73 to 0.85 | 0.83 to 0.88 | 91% to 97% | 0.74 to 0.85 | 0.83 to 0.87 |
| Note. *NICHD* = National Institute of Child Health and Human Development coding scheme; *PIIOS* = Parent -Infant Interaction Observation Scale; *ICC* = Intraclass Correlation Coefficient; *Gold* = Gold Standard Rater. | | | | | | | |
