# Supplementary material for: Identifying vulnerable mother-infant dyads: a psychometric evaluation of two observational coding systems using varying interaction periods
Source: Front Psychol. 2024 Jun 24;15:1399841. doi: 10.3389/fpsyg.2024.1399841 (PMC11233099; doi:10.3389/fpsyg.2024.1399841)
Supplement: Supplementary file 3 [file Table_3.DOCX]

Supplementary Material

**Table S3.** Area Under the Curve Analysis Results for Prediction of Child Mental Health Outcomes at Age 1 and 2 Based on NICHD (3 and 7 Minutes Observation)

|  | BITSEA predictor | 3 min  AUC | 7min  AUC |
| --- | --- | --- | --- |
| NICHD-3 score | Externalizing (age 1) | 0.73 | 0.76 |
|  | Internalizing (age 1) | 0.52 | 0.50 |
|  | Externalizing (age 2) | 0.63 | 0.74 |
|  | Internalizing (age 2) | 0.54 | 0.57 |
| NICHD Total score | Externalizing (age 1) | 0.72 | 0.73 |
|  | Internalizing (age 1) | 0.53 | 0.54 |
|  | Externalizing (age 2) | 0.64 | 0.67 |
|  | Internalizing (age 2) | 0.54 | 0.50 |
| Note. *NICHD* = National Institute of Child Health and Human Development coding scheme; *BITSEA* = Brief Infant Toddler Social-Emotional Scale | | | |
