# Supplementary material for: Identifying vulnerable mother-infant dyads: a psychometric evaluation of two observational coding systems using varying interaction periods
Source: Front Psychol. 2024 Jun 24;15:1399841. doi: 10.3389/fpsyg.2024.1399841 (PMC11233099; doi:10.3389/fpsyg.2024.1399841)
Supplement: Supplementary file 4 [file Table_4.DOCX]

Supplementary Material

**Table S4.** Area Under the Curve Analysis Results for Prediction of Child Mental Health at Age 1 and 2 Based on PIIOS (3 And 7 Minutes Observation)

|  | BITSEA predictor | 3 min  AUC | 7min  AUC |
| --- | --- | --- | --- |
| PIIOS domain score | Externalizing (age 1) | 0.57 | 0.60 |
|  | Internalizing (age 1) | 0.54 | 0.55 |
|  | Externalizing (age 2) | 0.73 | 0.70 |
|  | Internalizing (age 2) | 0.61 | 0.66 |
| PIIOS total score | Externalizing (age 1) | 0.64 | 0.68 |
|  | Internalizing (age 1) | 0.56 | 0.58 |
|  | Externalizing (age 2) | 0.78 | 0.77 |
|  | Internalizing (age 2) | 0.68 | 0.71 |
| Note. *PIIOS* = Parent -Infant Interaction Observation Scale; *BITSEA* = Brief Infant Toddler Social-Emotional Scale | | | |
