# Supplementary material for: Identifying vulnerable mother-infant dyads: a psychometric evaluation of two observational coding systems using varying interaction periods
Source: Front Psychol. 2024 Jun 24;15:1399841. doi: 10.3389/fpsyg.2024.1399841 (PMC11233099; doi:10.3389/fpsyg.2024.1399841)
Supplement: Supplementary file 5 [file Table_5.DOCX]

Supplementary Materials

**1** **Dimensions of parent-infant interaction within each observational coding system.**

## The NICHD-SECCYD coding scheme

Each scale is rated on a global 5-point scale, ranging from 1 (not at all characteristic) to 5 (highly characteristic).

### Parental Behavior is rated on the following scales (codes)

- Sensitivity to distress - captures the extent to which the mother responded to her infant’s cries, frets or distress in a consistent, timely, and appropriate manner.
- Sensitivity to non-distress - captures the extent to which the mother observed and responded in a well-paced and appropriate manner to her infant’s social gestures, expressions, and signals of non-distress.
- Global sensitivity - captures overall sensitivity to distress and non-distress episodes during play.
- Positive regard - captures the frequency and intensity of mother’s positive feelings expressed in behavior and quality of speech toward the child
- Negative regard - captures the mother’s negative regard for the child, both frequency and intensity of negative affect in behavior and speech towards the child are considered.
- Intrusiveness - captures the extent to which the interaction was adult centred rather than child centred, shown by behaviors such as not allowing the child to handle toys that they reach for or insisting that the child do something (play, eat, interact) in which they are not interested as well as physical intrusions such as constraining child’s movements, rough handling of the child, any other physical contact not welcomed by the child.
- Detachment – captures the extent to which the mother appeared emotionally uninvolved or disengaged and unaware of the child’s needs for appropriate interaction.
- Animation - captures mother’s level of animation, paying attention to aspects of behavior reflecting excitement, energy or interest in the interaction.
- Stimulation - captures the degree to which the mother tried to foster the child’s cognitive, motor or language development.

### Child Behavior is rated on the following scales (codes)

- Positive mood - captures the extent to which the child is content or pleased with the interaction.
- Negative mood - captures the frequency and intensity of child cries and fusses.
- Activity - captures how motorically active the child is throughout the interaction.
- Sustained attention - captures the child’s involvement with the physical world and rates how engaged the infant was within the interaction.

### Dyadic Behavior is rated on the following scale (code)

- Dyadic mutuality - captures the synchrony of interaction between mother and child and the amount of shared experience observed.

## The PIIOS coding scheme

Each dimension of the interaction is based on a 3-point categorical scale, assigning a score of 0 (no concern), 2 (some concern) or 4 (significant concern), with the lowest score indicating the most optimal interaction and the highest, the least optimal.

The 13 dimensions include:

- Infant positioning – captures infant’s ability to engage with caregiver, distance, stability and comfort.
- Eye contact – captures consistency of eye gaze between mother and infant.
- Vocalizations - both from mother and child.
- Affective engagement and synchrony - captures how appropriate the mother’s expression was and whether this matched up with the child’s mood.
- Warmth and affection – captures presence of genuine affection through mutual gazes and smiles.
- Holding and handling – captures whether or not the mother touched the child and the quality of touch, whether the mother was being too rough or the child appeared uncomfortable.
- Verbal commenting about or for the baby – captures whether or not the mother made positive or negative comments about the child or if they spoke for the child in a mind-minded manner.
- Attunement to distress - captures whether the mother responded sensitively to the infant’s cries or fusses.
- Bodily intrusiveness – captures presence of looming in or other physical intrusiveness.
- Expressed expectations about baby - captures the extent to which the mother had realistic and appropriate beliefs about their child’s developmental capabilities.
- Empathic understanding - captures the mother’s ability to understand their child’s intentions and reflect these back to the child.
- Responsive turn-taking – captures mother’s responsiveness to infant cues and synchrony in the interaction.
- Baby’s self-soothing strategies - whether or not the child engaged in self-comforting behavior in reaction to maternal intrusions and the amount of time they spent soothing.

# Exploratory Factor analysis for NICHD and PIIOS

Exploratory factor analysis was carried out to assess the latent constructs of the NICHD and PIIOS, for ordered categorical items, using the weighted least squares mean and variance adjusted (WLSMV) estimator (Muthén, du Toit, & Spisic, 1997). Measures of relative and absolute fit were used to assess the goodness of fit of the emerged structures. These measures and criteria were used: relative chi-square of values close to 2 indicate adequate fit (χ^2^/df; Hoelter, 1983), Root Mean Square Error of Approximation (RMSEA) values less than 0.05 are required for a close fit (Hu & Bentler, 1999), the root mean square residual (​RMR) (Hooper, Coughlan, & Mullen, 2008), the Tucker-Lewis Index (TLI; Bentler & Bonett, 1980) and the Comparative Fit Index (CFI; Hu & Bentler, 1999) both require values below 0.95 for close fit. Latent variable analysis was conducted using the Mplus software (Muthen & Muthén, 1998-2017).

This data driven approach was taken to determine if coding dimensions might form factors or subscales for each measure. Any such subscales might then be examined for their predictive validity in relation to the outcomes of interest in this study, alongside those selected on an a priori basis from the literature (e.g., NICHD-3, NICHD_total_, or, in the case of the PIIOS, PIIOS_total_ or PIIOS_domain_). To our knowledge previous published work on the psychometric properties of NICHD and PIIOS in the first year of life has not used factor analytic methods to aid scale construction.

## NICHD

The model which had good fit was a two-factor model which included the following codes: Global sensitivity, Detachment, Negative regard, Animation, Stimulation, Negative mood, Sustained attention, and Dyadic mutuality (Table S5). The remaining codes did not load in the model (Intrusiveness, Sensitivity to non-distress; Sensitivity to distress). The exercise was repeated for 3, 5 and 7 minutes of observation and the fit of the model was good in each instance (Table S6). Factor 1 represents Maternal and Dyadic Interaction Quality and factor 2 appeared to represent a latent composite of maternal and infant negativity and level of sustained infant attention during play (disengagement). With respect to reliability, high consistency emerged in the case of Factor 1 (Cronbach’s alpha about 0.9), item total correlations range (ITC) 0.58-0.78, inter item correlations range (IIC) 0.37-0.78. There were no problematic items in terms of alpha if item deleted (AID). Satisfactory indices emerged for Factor 2 (alpha about 0.6, ITC: 0.22-0.55, IIC: 0.28-0.50, no problematic items in terms AID) taking under consideration that it consists of only 3 items.

**Table S5.** Factor loadings for the EFA derived two-factor structure of the NICHD at the three durations.

|  | 3min | | 5min | | 7min | |
| --- | --- | --- | --- | --- | --- | --- |
| Codes | f1 | f2 | f1 | f2 | f1 | f2 |
| Detachment (M) | -0.99 |  | -0.94 |  | -0.96 |  |
| Positive regard (M) | 0.87 |  | 0.87 |  | 0.89 |  |
| Global sensitivity (M) | 0.81 |  | 0.83 |  | 0.78 |  |
| Animation (M) | 0.80 |  | 0.84 |  | 0.85 |  |
| Dyadic mutuality (M&C) | 0.76 | 0.44 | 0.78 | -0.36 | 0.76 | 0.36 |
| Stimulation (M) | 0.66 |  | 0.69 |  | 0.74 |  |
| Negative regard (M) |  | 0.42 |  | 0.50 |  | -0.50 |
| Negative mood (C) |  | 0.94 |  | 0.85 |  | -0.74 |
| Sustained attention (C) |  | -0.55 |  | -0.59 |  | 0.54 |
| *Note.* *f1*= Interaction Quality; *f2*= Negativity and infant disengagement; *M*=Maternal code, *C*=Child code. | | | | | | |

**Table S6.** Goodness of fit values for the EFA derived two-factor structure of the NICHD

| Duration | Rel χ^2^ | RMSEA | | | TLI | CFI | RMR |
| --- | --- | --- | --- | --- | --- | --- | --- |
|  |  |  | 90% CI | |  |  |  |
|  |  |  | *LL* | *UL* |  |  |  |
| 3 min | 2.92 | 0.088 | 0.061 | 0.120 | 0.98 | 0.99 | 0.051 |
| 5 min | 2.58 | 0.079 | 0.052 | 0.107 | 0.98 | 0.99 | 0.044 |
| 7 min | 2.80 | 0.085 | 0.058 | 0.112 | 0.98 | 0.99 | 0.047 |

## PIIOS

The PIIOS was found to have an acceptable fit as a two-factors model, for the 3- 5- and 7-minute observation (Table S7). In each model 11 out of the 13 coding dimensions loaded (exceptions were: Holding/Handling; Verbal Commenting). Factor one contains the codes of Vocalizations, Warmth and affection, Affective engagement and synchrony, Responsive turn-taking, Expressed expectations, Infant positioning, Empathic understanding, Eye contact and Attunement to distress. This factor measures Sensitivity/Responsiveness. Factor two contains Bodily intrusiveness (looming in) and Baby’s self-soothing strategies, perhaps reflecting frightening and frightened/distressed behaviors in mother and infant respectively (Table S8). With respect to reliability, satisfactory indices emerged in the case of Factor 1 (alpha about 0.8, ITC: 0.33-0.66, IIC: 0-0.73, no problematic items in terms of alpha if item deleted AID) and Factor 2 (alpha about 0.8, ITC: 0.64-0.71, IIC: 0.64-0.71, no problematic items in terms of alpha if item deleted AID).

**Table S7.** Goodness of fit values for the EFA derived two-factor structure of the PIIOS

| Duration | Rel χ^2^ | RMSEA | | | TLI | CFI | RMR |
| --- | --- | --- | --- | --- | --- | --- | --- |
|  |  |  | 90% CI | |  |  |  |
|  |  |  | *LL* | *UL* |  |  |  |
| 3 min | 2.86 | 0.086 | 0.066 | 0.107 | 0.953 | 0.971 | 0.082 |
| 5 min | 1.91 | 0.060 | 0.037 | 0.082 | 0.981 | 0.988 | 0.060 |
| 7 min | 1.91 | 0.060 | 0.037 | 0.082 | 0.982 | 0.989 | 0.057 |

**Table S8.** *Factor loadings of the EFA derived two-factor structure of the PIIOS*

|  | 3min | | 5min | | 7min | |
| --- | --- | --- | --- | --- | --- | --- |
| Codes | f1 | f2 | f1 | f2 | f1 | f2 |
| Vocalizations | 0.91 |  | 0.98 |  | 0.95 |  |
| Warmth and affection | 0.89 |  | 0.91 |  | 0.91 |  |
| Affective engagement and synchrony | 0.85 |  | 0.83 |  | 0.83 |  |
| Responsive turn-taking | 0.82 |  | 0.76 |  | 0.79 |  |
| Expressed expectations | 0.67 | 0.35 | 0.56 |  | 0.61 |  |
| Infant positioning | 0.62 |  | 0.62 |  | 0.60 |  |
| Empathic understanding | 0.62 |  | 0.50 | 0.30 | 0.57 |  |
| Eye contact | 0.56 |  | 0.62 |  | 0.64 |  |
| Attunement to distress | 0.51 |  | 0.54 |  | 0.56 |  |
| Bodily intrusiveness (looming in) |  | 1.00 |  | 1.06 |  | 1.04 |
| Baby's self-soothing strategies |  | 0.83 |  | 0.75 |  | 0.81 |
| *Note.* *f*1 reflects Sensitivity; *f*2 reflects Frightening and frightened/distressed maternal and infant behavior | | | | | | |

ROC curve analyses using Factor scores derived from NICHD and PIIOS as predictors of Attachment and Mental Health Outcomes at age 1 and 2.

The four tables (S9 to S12) below show poor discriminant validity for the NICHD and PIIOS Factors scores for each of the Attachment and mental health outcomes at age 1 and 2 and for each observation period with AUC’s <.70.

**Table S9.** Area under the curve for secure attachment prediction based on NICHD and PIIOS Factor Scores.

| Predictor | 3 min  AUC | 5min  AUC | 7min  AUC | ΔAUC | |
| --- | --- | --- | --- | --- | --- |
|  |  |  |  | *χ^2^* (df) | *p* |
| NICHD Factor 1 score | 0.62 | 0.64 | 0.64 | 3.83 (2) | 0.147 |
| NICHD Factor 2 score | 0.58 | 0.59 | 0.57 | 1.13 (2) | 0.568 |
| PIIOS Factor 1 score | 0.41 | 0.41 | 0.41 | 0.18 (2) | 0.914 |
| PIIOS Factor 2 score | 0.50 | 0.51 | 0.52 | 1.14 (2) | 0.566 |

**Table S10.** Area under the curve for disorganized attachment prediction based on NICHD and PIIOS Factor Scores.

| Predictor | 3 min | 5min | 7min | ΔAUC | |
| --- | --- | --- | --- | --- | --- |
|  | AUC | AUC | AUC | χ^2^ (df) | p |
| NICHD Factor 1 score | 0.56 | 0.56 | 0.56 | 0.39 (2) | 0.821 |
| NICHD Factor 2 score | 0.52 | 0.50 | 0.49 | 1.19 (2) | 0.550 |
| PIIOS Factor 1 score | 0.54 | 0.52 | 0.52 | 1.59 (2) | 0.452 |
| PIIOS Factor 2 score | 0.49 | 0.47 | 0.47 | 1.26 (2) | 0.533 |

**Table S11.** Area under the curve for BITSEA prediction based on NICHD Factor Scores.

| Predictor | Outcome | 3 min | 5min | 7min | ΔAUC | |  |
| --- | --- | --- | --- | --- | --- | --- | --- |
|  | BITSEA | AUC | AUC | AUC | *χ^2^* (df) | *p* | |
| NICHD Factor 1 | Externalizing (age 1) | 0.56 | 0.53 | 0.56 | 3.98 (2) | 0.137 | |
|  | Internalizing (age 1) | 0.50 | 0.49 | 0.51 | 1.56 (2) | 0.458 | |
|  | Externalizing (age 2) | 0.54 | 0.54 | 0.54 | 0.07 (2) | 0.964 | |
|  | Internalizing (age 2) | 0.46 | 0.48 | 0.49 | 0.56 (2) | 0.757 | |
| NICHD Factor 2 | Externalizing (age 1) | 0.46 | 0.47 | 0.50 | 2.11 (2) | 0.348 | |
|  | Internalizing (age 1) | 0.44 | 0.44 | 0.45 | 0.48 (2) | 0.787 | |
|  | Externalizing (age 2) | 0.54 | 0.61 | 0.59 | 5.97 (2) | 0.051 | |
|  | Internalizing (age 2) | 0.53 | 0.58 | 0.56 | 1.82 (2) | 0.402 | |

**Table S12.** Area under the curve for BITSEA prediction based on PIIOS Factor Scores

|  | Outcome  BITSEA | 3 min AUC | 5min  AUC | 7min  AUC | ΔAUC | |  |
| --- | --- | --- | --- | --- | --- | --- | --- |
|  |  |  |  |  | *χ^2^* (df) | *p* | |
| PIIOS Factor 1 | Externalizing (age 1) | 0.54 | 0.54 | 0.54 | 0.04 (2) | 0.982 | |
|  | Internalizing (age 1) | 0.53 | 0.54 | 0.54 | 0.08 (2) | 0.959 | |
|  | Externalizing (age 2) | 0.63 | 0.67 | 0.63 | 5.61 (2) | 0.060 | |
|  | Internalizing (age 2) | 0.62 | 0.67 | 0.66 | 3.74 (2) | 0.154 | |
| PIIOS Factor 2 | Externalizing (age 1) | 0.45 | 0.50 | 0.54 | 2.18 (2) | 0.335 | |
|  | Internalizing (age 1) | 0.47 | 0.49 | 0.50 | 0.30 (2) | 0.863 | |
|  | Externalizing (age 2) | 0.62 | 0.61 | 0.58 | 0.89 (2) | 0.640 | |
|  | Internalizing (age 2) | 0.58 | 0.59 | 0.57 | 0.22 (2) | 0.896 | |

# Regularized regression and Machine Learning Analysis

Regularized methods (Lasso Regression) and cross-validation (Machine Learning) were used to examine the prediction of attachment classification and symptom outcomes. Both the NICHD and PIIOS coding schemes provide ratings of a range of behaviors, some principally of the mother, some of the child and some that are intrinsically dyadic in nature. It is possible that there are particular coding dimensions and combinations of those dimensions that might be of particular salience for later development of secure attachment or behavioral problems. Such a search requires choosing from among a large number of potential prediction models and requires special attention being paid to the problems of overfitting. Overfitting leads to coefficient estimates being upwardly biased in magnitude, and an exaggerated assessment of prediction success. We applied lasso and split-sample cross-validation methods to reduce the extent of these biases. We compared the performance of the two coding schemes using several sets of predictors in each case.

## A priori

The first was the single item (appropriately signed) sum score from each scale. There being no model selection this used regular logistic regression informed by the literature. In the case of the PIIOS the total score (PIIOS_total_) was used as the instrument was developed with the intention of a total score being derived to reflect overall quality of interaction. In the case of NICHD, the literature has typically focused on ratings derived from combinations of the 7 adult scales (Global sensitivity, detachment (reversed), intrusiveness (reversed), animation, stimulation, positive regard, negative regard (reversed) AND one dyadic scale (dyadic mutuality) to predict child development outcomes such as behavioral problems. Accordingly, we selected to examine prediction to total behavioral problems at age 1 and 2 from (i) a total score representing all 8 NICHD dimensions (NICHD_total_) of adult behavior including dyadic mutuality and (ii) a commonly used trio of codes (NICHD-3); Global sensitivity, intrusiveness (Reversed) and Positive Regard), identified below as the minimum set.

## A priori 3

The set of 3 NICHD items (NICHD-3), global sensitivity, intrusiveness and positive regard most commonly identified in the literature. There is no equivalent evidence base for PIIOS.

## Main

This set included all the individual item scores as main effects and likely freely available demographics (maternal age, child sex, IMD deprivation quintile associated with postcode, maternal education beyond school, marital status).

## Interaction

This set included both main effects included in Main but also all pairwise (two-way) interactions, for example allowing for the possibility that maternal intrusiveness in a context of low maternal warmth might be of particular concern.

Table S13 shows the numbers of variables selected from the available pool that achieved the highest cross-validation and the corresponding Deviance Ratios (DR) within the training and validation samples. Higher DR indicates better prediction performance and lower DR in the validation sample compared to the training sample indicates overfitting and poor generalization of the model. The left hand column identifies the increasingly large sets of prediction variables: **A priori**  - the NICHD_total_ alone or PIIOS_total_, **A priori 3** – proposed subset of items (NICHD-3 only), **Main** – all items and available background variables (25 NICHD and 24 PIIOS) and **Interactions** – all items and background with interactions (129 NICHD and 115 PIIOS in all). We see that models where large numbers of variables were selected from the available predictors into the “best” model perform well on the target sample but very poorly on the validation sample. The highest deviance ratios estimated by cross-validation came from use of the *a priori* specified item totals. We take these models as the benchmark. These show modest predictive power in the validation sample for Age 1 secure attachment, somewhat weaker for externalizing and weaker still for internalizing behavior at Age 1. Examining prediction models using all the individual items and background variables proved to perform worse than the benchmarks. This also proved the case when examining all the possible two-way item interactions.

**Table S13.** Determining whether the Interaction Ratings Could Predict in New Samples- Deviance ratios (analogous to R-squared) from 3:1 training-validation split sample 10-fold cross-validation

|  | Secure Attachment | | | BITSEA Externalizing Age 1 | | | BITSEA Internalizing Age 1 | | |  |
| --- | --- | --- | --- | --- | --- | --- | --- | --- | --- | --- |
|  | N of predictors | Training n=196 | Validation n=69 | N of predictors | Training n=188 | Validation n=64 | N of predictors | Training n=188 | Validation n=64 |  |
| NICHD |  |  |  |  |  |  |  |  |  |  |
| A priori 8 | 1 of 1 | 0.152 | 0.230 | 1 of 1 | 0.244 | 0.139 | 1 of 1 | 0.169 | 0.232 |  |
| A priori 3 | 1 from 1 | 0.177 | 0.322 | 1 from 1 | 0.24 | 0.140 | 1 from 1 | 0.161 | 0.220 |  |
| Main (lasso) | 8 from 25 | 0.125 | -0.024 | 15 from 25 | 0.419 | -0.395 | 0 from 25 | 0.000 | -0.005 |  |
| Interactions (lasso) | 12 from 129 | 0.235 | 0.130 | 13 from 129 | 0.384 | -0.302 | 0 from 129 | 0.000 | -0.005 |  |
| PIIOS |  |  |  |  |  |  |  |  |  |  |
| A priori | 1 of 1 | 0.157 | 0.320 | 1 of 1 | 0.233/ | 0.098 | 1 of 1 | 0.182 | 0.228 |  |
| Main (lasso) | 6 from 24 | 0.095 | -0.059 | 7 from 24 | 0.169 | -0.078 | 1 from 24 | 0.268 | 0.090 |  |
| Interactions (lasso) | 2 from 115 | 0.090 | -0.085 | 4 from 115 | 0.158 | -0.074 | 2 from 115 | 0.013 | 0.005 | |

Table S14 shows results for BITSEA outcomes at age 2 separated for internalizing and externalizing problems. Again, the simple A priori item composites nearly always perform best in the validation sample.

**Table S14.** Determining whether the Interaction Ratings Could Predict in New Samples- Deviance ratios (analogous to R-squared) from 3:1 training-validation split sample 10-fold cross-validation (training over validation)

|  | BITSEA Externalizing Age 2 | | BITSEA Internalizing Age 2 | |
| --- | --- | --- | --- | --- |
|  | Training | Validation | Training | Validation |
| NICHD |  |  |  |  |
| A priori 8 | 0.152 | 0.230 | 0.218 | 0.106 |
| A priori 3 | 0.177 | 0.322 | 0.194 | 0.111 |
| Main (lasso) | 0.125 | -0.024 | -0.002 | -0.007 |
| Interactions (lasso) | 0.235 | 0.130 | 0.193 | -0.130 |
| PIIOS |  |  |  |  |
| A priori | 0.214 | 0.042 | 0.235 | 0.048 |
| Main (lasso) | 0.157 | -0.475 | -0.002 | -0.068 |
| Interactions (lasso) | 0.157 | -0.068 | -0.002 | -0.068 |

# References for Supplementary Material

Bentler, P. M., & Bonett, D. (1980). Significance tests and goodness of fit in the analysis of covariance structures. Psychol. Bull. 88, 588-606. doi: 10.1037/0033-2909.88.3.588

Hoelter, J. (1983). The analysis of covariance structures. Sociol. Methods Res. 11, 325-344. doi: 10.1177/0049124183011003003

Hooper, D., Coughlan, J., & Mullen, M.R. (2008). Structural equation modelling: Guidelines for determining model fit. J. Bus. Res. Methods, 6, 53–60. doi: 10.21427/D7CF7R

Hu, L., & Bentler, P. (1999). Cutoff criteria for fit indexes in covariance structure analysis: Conventional criteria versus new alternatives. Structural Equation Modeling: A Multidisciplinary Journal, 6, 1-55. doi: 10.1080/10705519909540118

Muthén, B., du Toit, S. H. C., & Spisic, D. (1997). Robust inference using weighted least squares and quadratic estimating equations in latent variable modelling with categorical and continuous outcomes. https://www.statmodel.com/download/Article_075.pdf

Muthen, L. K., & Muthén, B. (1998-2017). Mplus User’s Guide (8th ed).
